# Knockdown of *Tlr4* in the Arcuate Nucleus Improves Obesity Related Metabolic Disorders

**DOI:** 10.1038/s41598-017-07858-6

**Published:** 2017-08-07

**Authors:** Yongli Zhao, Guohua Li, Ying Li, Yuchuan Wang, Zhengjuan Liu

**Affiliations:** Department of Pediatrics, The Second Hospital of Dalian Medical University, 467 Zhongshan Road, Shahekou District, Dalian, 116027 Liaoning China

## Abstract

High-fat diet-induced hypothalamic metabolic inflammation is emerging as a cause for the development of obesity. It is acknowledged that Toll-like receptor4 (TLR4) signaling plays a crucial role in triggering of the hypothalamic metabolic inflammation during the course of diet-induced obesity. Whether hypothalamic arcuate nucleus (ARC)-restricted TLR4 knockdown improves obesity-related metabolic disorders remains unexplored. In this study, we used TLR4 shRNA lentiviral particles to suppress the TLR4 expression in the hypothalamic ARC of diet-induced obese rat model by stereotaxic injection. Our results demonstrate that ARC-restricted TLR4 knockdown protects obese rats from diet-induced weight gain and energy intake, from diet-induced impaired glucose homeostasis and peripheral insulin resistance, and from high-fat diet-induced hepatic steatosis and adipocyte hypertrophy. Thus, we define ARC-restricted TLR4 knockdown as a potential strategy to combat metabolic disorders associated with obesity.

## Introduction

The World Health Organization (WHO) has estimated that the worldwide prevalence of obesity has doubled between 1980 and 2014^1^. Adults with obesity have a high chance of getting some devastating diseases (type 2 diabetes, cardiovascular disease, *et al*.)^1^; children with obesity could experience breathing difficulties, increased risk of fractures, and psychological effects, *et al*.^1^. It is well known that the fundamental cause of obesity is the imbalance of energy homeostasis system, which includes energy intake and expenditure^2^. However, the effect of caloric restriction and exercise on weight loss is inadequate, and the lost weight is easily regained^2^. Therefore, it is necessary to investigate approaches that are more effective for treatment of obesity.

The arcuate nucleus (ARC), which sits adjacent to the third ventricle in the mediobasal hypothalamus (MBH), contains two groups of neurons; one group is orexigenic and releases the neurotransmitters neuropeptide Y (NPY) and agouti-related peptide (AgRP) (NPY/AgRP neurons). The other is anorexigenic and contains neurons expressing proopiomelanocortin (POMC neurons). These two types of neurons can mutually regulate, interact, and form a neuronal feedback circuit, which is the basic regulatory mechanism of energy homeostasis in hypothalamus^3, 4^. Numerous studies have suggested that the dysfunction of the arcuate nucleus energy balance circuit is associated with the majority of diet-induced obesity^5^, and revealed that diet-induced hypothalamic inflammation might play a crucial role in leading dysfunction of the neuronal circuitry^6, 7^. The toll-like receptor-4 (TLR4) is a member of the interleukin-1 receptor superfamilies that belong to pattern recognition receptors, and has a prominent role in activation of innate immune responses^8^. In the hypothalamic ARC, TLR4 is predominantly expressed in microglia^9, 10^. Recent study suggested that activation of TLR4 signaling in hypothalamus could trigger hypothalamic inflammatory response and lead to the resistance to anorexigenic signals, which has a crucial role in the genesis of obesity^10^. Another study revealed that activation of TLR4 by acute exposure to lipopolysaccharide (LPS) could affect the ARC neuronal activity and feeding behavior^9^. Milanski *et al*. found that inhibition of hypothalamic TLR4 by intracerebroventricular injection of anti-TLR4 antibody improved hepatic insulin signal transduction and reduced steatosis and gluconeogenesis^11^. These studies suggest that TLR4 in hypothalamus might be an attractive target for therapeutics of diet-induced obese condition.

In this study, we investigate whether down-regulation of TLR4 expression in hypothalamic ARC by stereotaxic injection of TLR4 shRNA lentiviral particles can improve the metabolic disorders in high-fat diet-induced obesity rat model.

## Results

### The expression of *Tlr4* mRNA in the hypothalamic ARC was down regulated by *Tlr4* shRNA lentiviral particles

**Figure 1:**
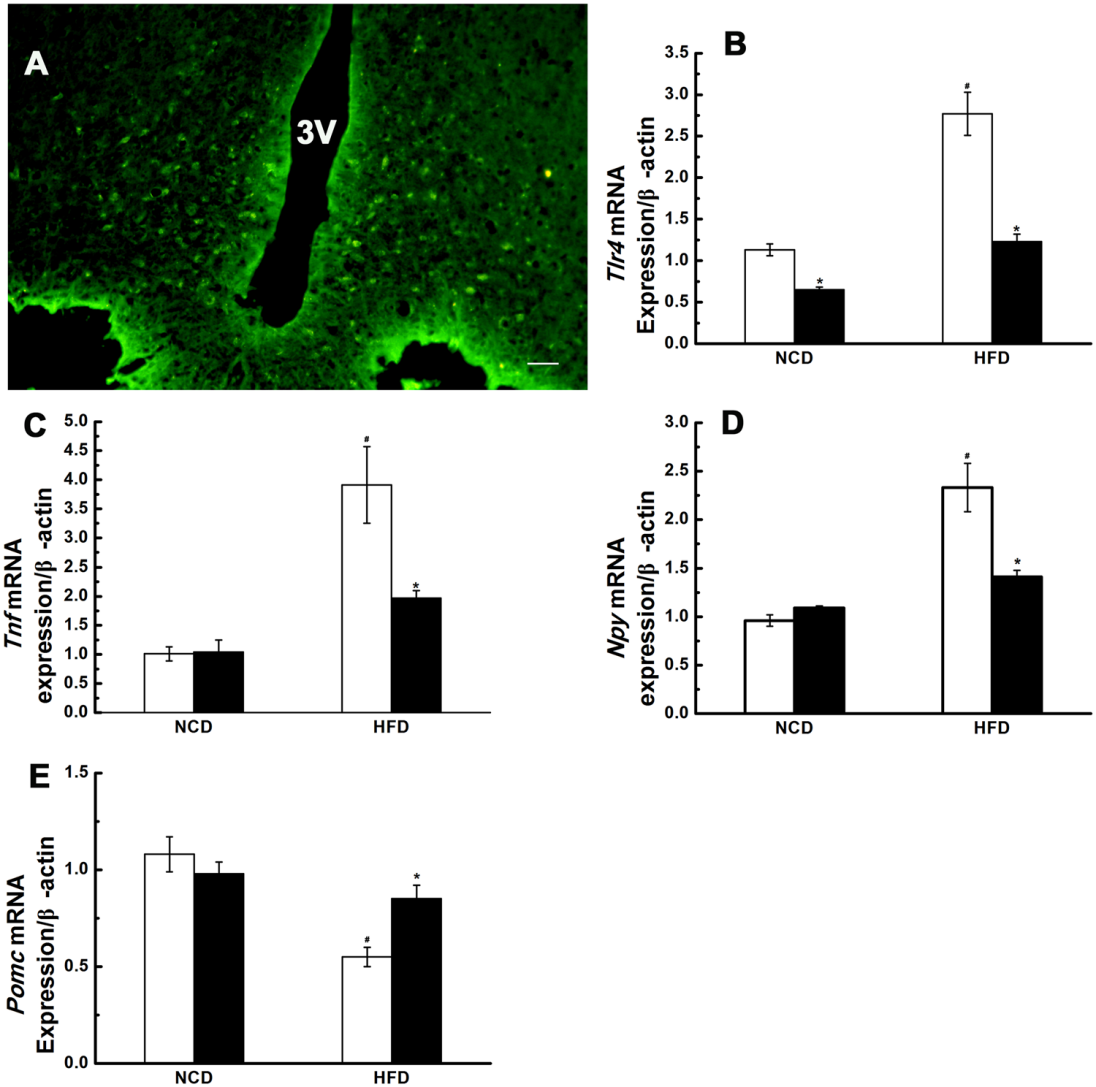
The mRNA expression of *Tlr4*, *Tnf, Npy* and *Pomc* in the hypothalamic arcuate nucleus (ARC) at the 4th week after stereotaxical injection of *Tlr4* shRNA lentiviral particles or scrambled shRNA lentiviral particles. (**A**) The copGFP Control Lentiviral Particles was used to examine the transduction efficiency in hypothalamic ARC at the 4th week after stereotaxic injection. Fluorescent microscopic image showed GFP-positive cells were still detected in the ARC after 4 weeks of injection. Scale bar, 100 μm. The expression of *Tlr4* mRNA (**B**), *Tnf* mRNA(**C**), *Npy* mRNA (**D**) and *Pomc* mRNA (**E**) in the ARC were detected by real-time RT-PCR. The expression level of mRNA was normalized to the b-actin values. The white bar represents *scrambled* shRNA treated group, and the black bar represents *Tlr4* shRNA treated group. NCD: normal chow diet group; HFD: high fat diet group; 3 V: the third ventricle. n = 7–8 per group. The results are shown as the mean ± S.E.M. ^#^P < 0.05 for HFD group compared with NCD group; *P < 0.05 for *Tlr4* shRNA treated group compared with *scrambled* shRNA treated group.

At the 4th week after stereotaxical injection of *Tlr4* shRNA lentiviral particles, the expression of *Tlr4* mRNA in the hypothalamic ARC was measured by real time RT-PCR. The expression of *Tlr4* mRNA was reduced by 42% in NCD group, and 56% in HFD group respectively (Fig. 1B). The copGFP Control Lentiviral Particles was used to examine the transduction efficiency of the lentiviral particles in the hypothalamic ARC. Fluorescent microscopic image showed GFP-positive cells were still detected in the hypothalamic ARC after 4 weeks of injection (Fig. 1A).

### Down-regulation of *Tlr4* mRNA expression in the hypothalamic ARC reduced hypothalamic inflammation and *Npy* expression, and reversed *Pomc* expression

High-fat diet induced significant inflammation in the hypothalamic ARC (P < 0.05, Fig. 1C), and prompted the activation of NPY/AgRP neurons (P < 0.05, Fig. 1D), and inhibited the activation of POMC neurons (P < 0.05, Fig. 1E). Treatment with *Tlr4* shRNA lentiviral particles by stereotaxical injection significantly reduced the mRNA expression of *Tnf* and *Npy*, and reversed *Pomc* expression in the hypothalamic ARC of obese rats (P < 0.05, Fig. 1C,D and E). However, it had no significant effect on the expression of *Tnf*, *Npy* and *Pomc* in the hypothalamic ARC of NCD group (P > 0.05, Fig. 1C,D and E).

### Down-regulation of *Tlr4* mRNA expression in the hypothalamic ARC reduced body weight and energy intake in obese rats

**Figure 2:**
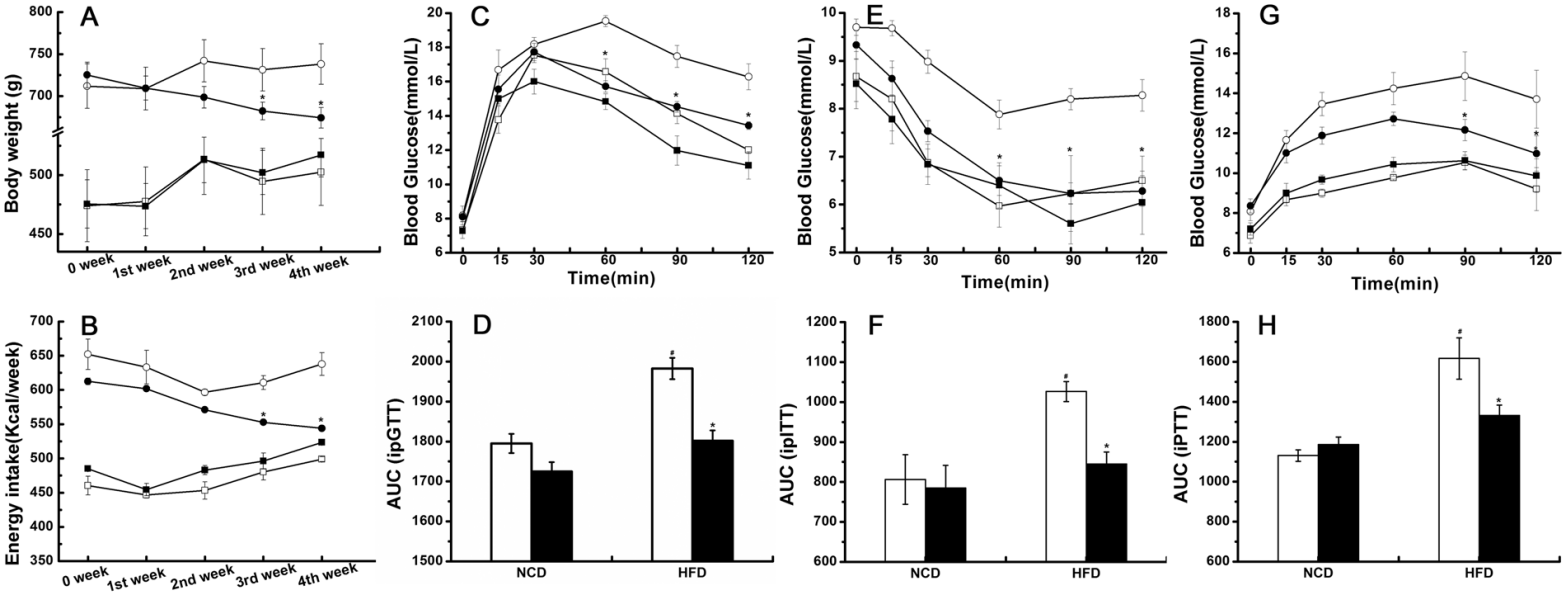
Down-regulation of *Tlr4* mRNA expression in the hypothalamic ARC decreases body weight, energy intake, and improves glucose tolerance, insulin sensitivity and liver gluconeogenesis in obese rats. (**A**) The changes of the body weight in NCD group and HFD group after treatment with *Tlr4* shRNA or scrambled shRNA lentiviral particles. (**B**) The changes of the average food intake in NCD group and HFD group after treatment with *Tlr4* shRNA or *scrambled* shRNA lentiviral particles. (**C**) The results of intraperitoneal glucose tolerance test (ipGTT) and (**D**) respective areas under the curve (AUC). (**E**) The results of intraperitoneal insulin tolerance test (ipITT) and (**F**) respective areas under the curve (AUC). (**G**) The results of intraperitoneal pyruvate tolerance test (iPTT) and (**H**) respective areas under the curve (AUC). The white circles and squares respectively represent treatment of *scrambled* shRNA in HFD group and NCD group; the black circles and squares respectively represent treatment of *Tlr4* shRNA in HFD group and NCD group. The white bar represents scrambled shRNA treated group, and the black bar represents *Tlr4* shRNA treated group. NCD: normal chow diet group; HFD: high fat diet group; n = 7–8 per group. The results are shown as the mean ± S.E.M. ^#^P < 0.05 for HFD group compared with NCD group; *P < 0.05 for *Tlr4* shRNA treated group compared with *scrambled* shRNA treated group.

At the 0 week, the HFD-induced obese rats were randomly divided into two groups (HFD + *Scrambled* shRNA and HFD + *Tlr4* shRNA). There were no significant difference in average body weight between the two groups (711.88 ± 26.58 g vs 725.13 ± 15.18 g, P > 0.05). There were also no significant difference in average energy intake between the two groups (652.15 ± 22.14 g vs 612.54 ± 4.69 g, P > 0.05). When HFD-induced obese rats were treated with *Tlr4* shRNA lentiviral particles by stereotaxical injection, their body weight and energy intake were significantly decreased during the 4-week observation period (Fig. 2A and B). At the final week (4th week), the average body weight of HFD + *Tlr4* shRNA group was significantly decreased than that of HFD + *Scrambled* shRNA group (674.25 ± 12.21 g vs 738.13 ± 24.04 g, P < 0.05). The average energy intake was also significantly decreased in HFD + *Tlr4* shRNA group than in HFD + *Scrambled* shRNA group (543.95 ± 1.09 vs 637.96 ± 16.51, P < 0.05). However, stereotaxical injection of *Tlr4* shRNA lentiviral particles had no significant effect on the body weight and energy intake in NCD group during the 4-week observation period (P > 0.05, Fig. 2A and B).

### Down-regulation of *Tlr4* mRNA expression in the hypothalamic ARC improved glucose homeostasis, insulin sensitivity and abnormal gluconeogenesis in obese rats

The glucose homeostasis was determined by intraperitoneal glucose tolerance test. The results revealed that treatment with *Tlr4* shRNA lentiviral particles significantly corrected abnormal glucose homeostasis in HFD-fed rats (P < 0.05, Fig. 2C and D). The insulin sensitivity was determined by intraperitoneal insulin tolerance test. The results revealed that treatment with *Tlr4* shRNA lentiviral particles significantly restored the insulin responsiveness in HFD-fed rats (P < 0.05, Fig. 2E and F). The intraperitoneal pyruvate tolerance test was used to determine the gluconeogenesis in obese rats. The obesity related abnormal gluconeogenesis was significantly ameliorated by down-regulation of *Tlr4* mRNA expression (P < 0.05, Fig. 2G and H). However, down-regulation of *Tlr4* mRNA expression did not affect the glucose homeostasis, insulin sensitivity and gluconeogenesis in NCD group (P > 0.05, Fig. 2C–H).

### Down-regulation of *Tlr4* mRNA expression in the hypothalamic ARC ameliorated periphery blood metabolic disorders and inflammation in obese rats

The fasting glucose, FFA and triglyceride in periphery blood were significantly increased in HFD-fed rats than in NCD-fed rats (Table 2). These obese rats also manifested significant hyperinsulinemia and hyperleptindemia (Table 2). High fat diet also induced higher levels of inflammatory markers (TNF-αand IL-6) in periphery blood of obese rats (Table 2). The obesity related metabolic disorders and inflammation were ameliorated after down-regulation of *Tlr4* mRNA expression, excluding the fasting glucose and triglyceride (Table 2). However, down-regulation of *Tlr4* mRNA expression did not affect these metabolic and inflammatory markers in NCD group (Table 2).

### Down-regulation of *Tlr4* mRNA expression in the hypothalamic ARC reversed hepatic steatosis and adipocyte hypertrophy

**Figure 3:**
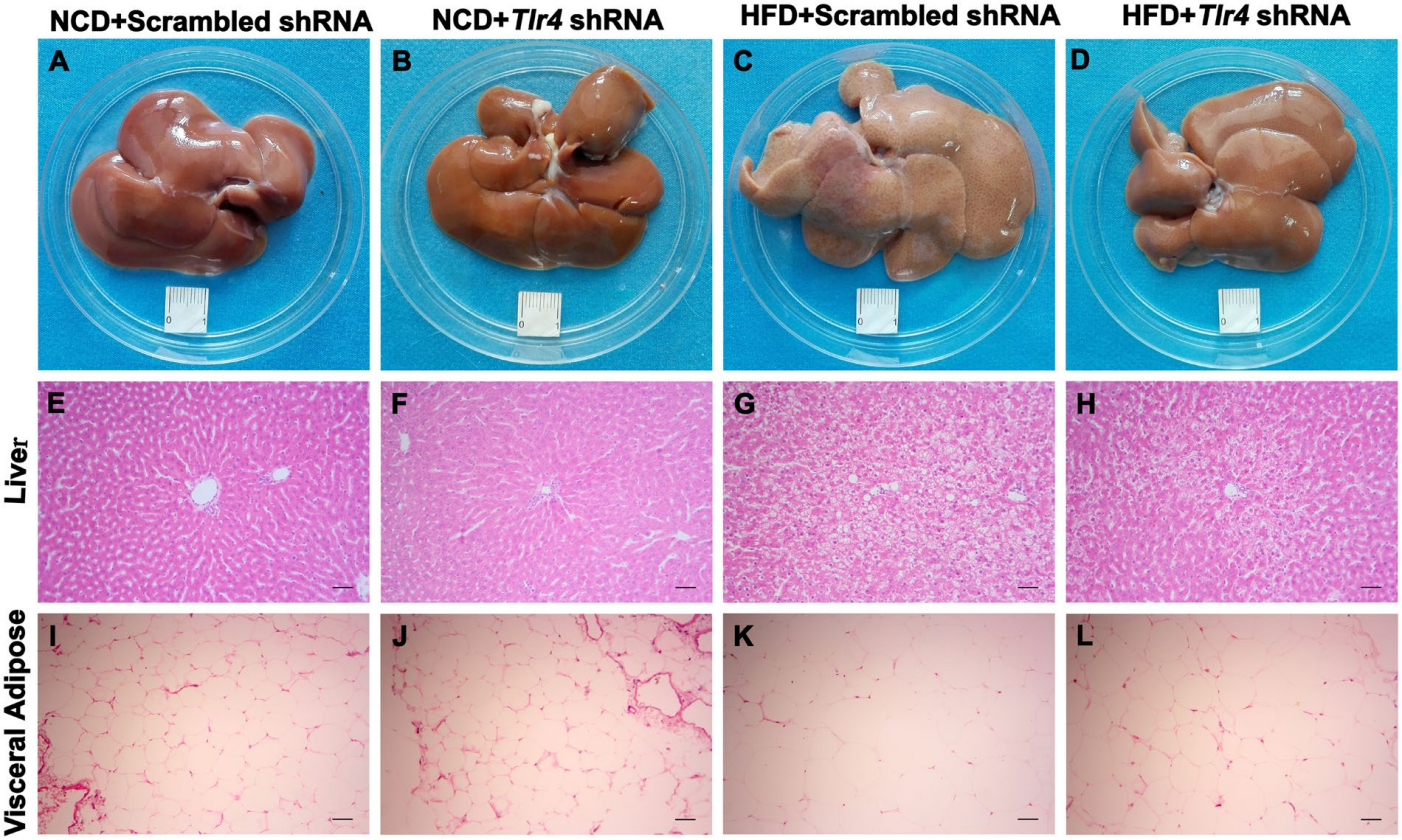
Down-regulation of *Tlr4* mRNA expression in the hypothalamic ARC improves hepatic steatosis and adipocyte hypertrophy. (**A**–**D**) Macroscopic pictures of the livers of rats in each group indicated. Scale bar, 1 cm. (**E**–**H**) Histological analysis of liver stained with hematoxylin and eosin in the groups indicated. Scale bars, 100 μm. (**I**–**L**) Histological analysis of visceral adipose stained with hematoxylin and eosin in the groups indicated. Scale bars, 100 μm. NCD: normal chow diet group; HFD: high fat diet group.

The typical hepatomegaly was observed in HFD-fed rats (Fig. 3C). The average liver weight was significantly increased in HFD group than in NCD group (P < 0.05, Table 2). Histological analysis revealed that large lipid droplets were widespread in the livers from obese rats (Fig. 3G). These changes of gross morphology and histopathology in the livers from obese rats were partially reversed after down-regulation of *Tlr4* mRNA expression (Fig. 3D and H). However, down-regulation of *Tlr4* mRNA expression did not affect the livers from NCD group (Fig. 3A,B,E and F) (Table 2). In addition, accompanied with hepatic steatosis, the level of the liver function marker, aspartate aminotransferase (AST), was significantly increased in the periphery blood from obese rats (P > 0.05, Table 1). Down-regulation of *Tlr4* mRNA expression could not ameliorate this change (Table 2).

**Table 1:** The sequences of the primer used in real-time RT-PCR.

Gene	Forward Primer	Reverse Primer
*Tlr4*	5′-CCGCTCTGGCATCATCTTCA-3′	5′-CCCACTCGAGGTAGGTGTTTCTG-3′
*Npy*	5′-CCGCTCTGCGACACTACATC-3′	5′-GGGCATTTTCTGTGCTTTCTCT-3′
*Pomc*	5′-GCTACGGCGGCTTCATGA-3′	5′-CCTCACTGGCCCTTCTTGTG-3′
*Ccl2*	5′-CTCTTTTCCACAACCACCTCAA-3′	5′-GGCATCACATTCCAAATCACA-3′
*Tnf*	5′-GGCGTGTTCATCCGTTCTC-3′	5′-CTTCAGCGTCTCGTGTGTTTCT-3′
*Pklr*	5′-ATCTGGGCAGATGATGTGGA-3′	5′-ATAGGGTGTAACTGGGTCAGAATGG-3′
*G6pc*	5′-AACGTCTGTCTGTCCCGGATCTA-3′	5′-CCTCTGGAGGCTGGCATTGTA-3′
*Fasn*	5′-GCTGCTACAAACAGGACCATCAC-3′	5′-TCTTGCTGGCCTCCACTGAC-3′
*Acaca*	5′-CAATCCTCGGCACATGGAGA-3′	5′-GCTCAGCCAAGCGGATGTAGA-3′
*β-actin*	5′-CCTAAGGCCAACCGTGAAAA-3′	5′-CAGAGGCATACAGGGACAACAC-3′

**Table 2:** Biochemical parameters of rats in the groups indicated at the 4th week after stereotaxical injection of Tlr4 shRNA or Scrambled shRNA lentiviral particles.

	NCD		HFD	
	*Scrambled* shRNA	*Tlr4* shRNA	*Scrambled* shRNA	*Tlr4* shRNA
Fasting Glucose (mmol/L)	6.94 ± 0.11	7.28 ± 0.09	8.78 ± 0.32^#^	8.24 ± 0.31
FFA (μmol/L)	289.38 ± 14.88	290.52 ± 21.59	321.1 ± 37.05^#^	299.51 ± 23.77^*^
TNF-α (pg/ml)	3.35 ± 0.17	3.39 ± 0.51	7.03 ± 0.31^#^	5.74 ± 0.28^*^
IL-6 (pg/ml)	2.93 ± 0.32	2.97 ± 0.19	5.24 ± 0.23^#^	3.47 ± 0.18^*^
Insulin (mIU/L)	12.75 ± 0.33	13.6 ± 0.45	28.99 ± 1.45^#^	18.54 ± 0.29^*^
Leptin (ng/ml)	6.53 ± 0.62	6.66 ± 0.98	13.2 ± 0.66^#^	9.26 ± 0.53^*^
Triglyceride (mmol/l)	0.66 ± 0.05	0.64 ± 0.1	1.39 ± 0.14^#^	1.27 ± 0.1^*^
Cholesterol (mmol/l)	1.48 ± 0.19	1.50 ± 0.16	1.36 ± 0.14	1.39 ± 0.09
AST (U/L)	92.20 ± 14.7	86.80 ± 13.78	145.6 ± 10.33^#^	140 ± 7.73
ALT (U/L)	28.20 ± 2.65	27.40 ± 3.26	25.2 ± 2.84	21.6 ± 2.18
Liver Weight (g)	15.99 ± 0.94	16.53 ± 0.87	23.34 ± 0.58^#^	19.46 ± 0.41^*^
Visceral Adipose (g)	22.21 ± 2.46	21.27 ± 2.2	52 ± 5.04^#^	36.38 ± 2.91^*^

Abbreviation: FFA, Free fatty acids; TNF-α, Tumor Necrosis Factor α; IL-6, Interluekin-6; AST, Aspartate Aminotransferase; ALT, Alanine aminotransferase; NCD, Normal chow diet; HFD, High fat diet. All data in the table are shown as the mean ± S.E.M. (n = 7–8/group). *P < 0.05: HFD + *Tlr4* shRNA group vs HFD + *Scrambled* shRNA; ^#^ *P* < 0.05: HFD group vs NCD group.

**Figure 4:**
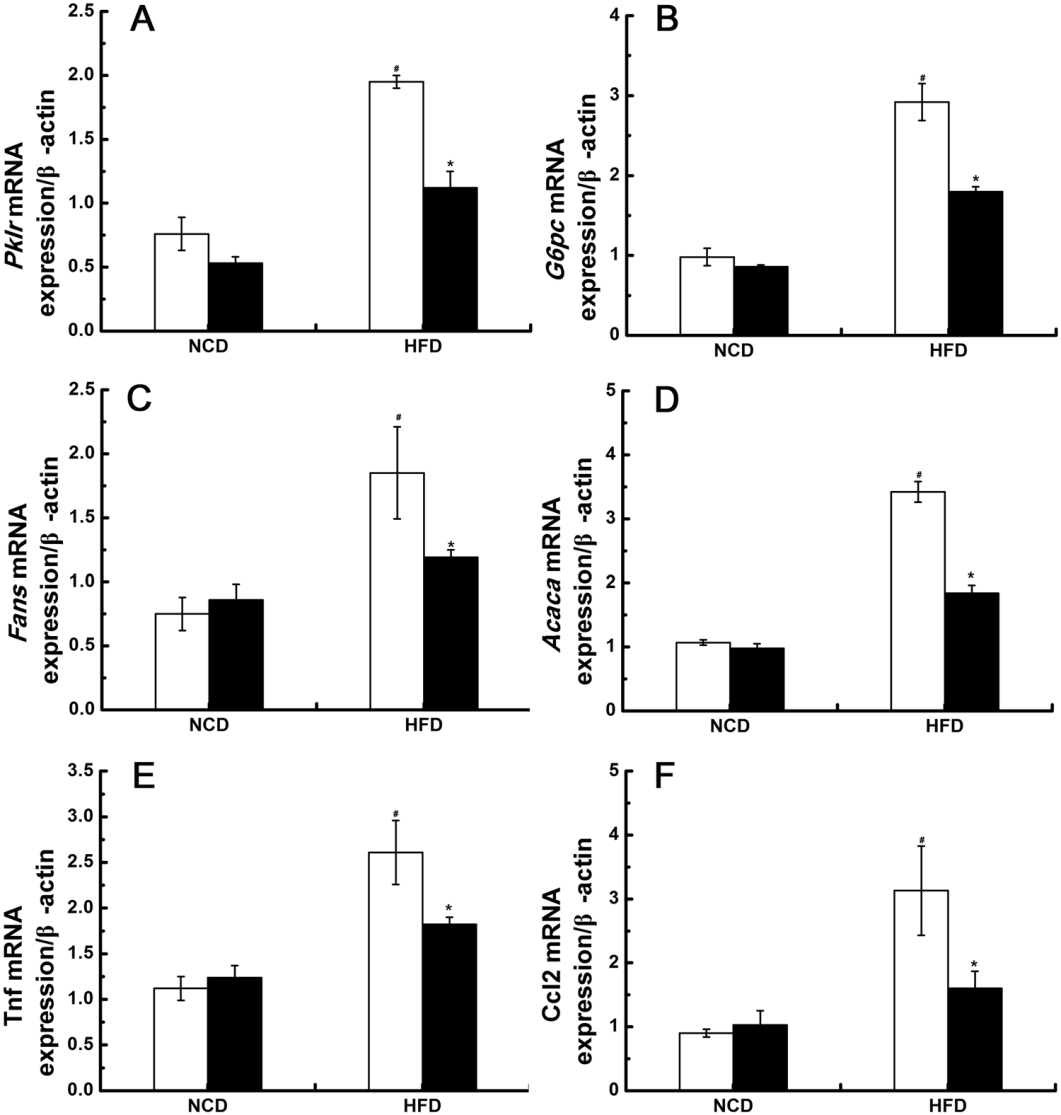
Down-regulation of *Tlr4* mRNA expression in the hypothalamic ARC improves the metabolic parameters and inflammation in liver. (**A** and **B**) the changes of glucose metabolism genes (*Pklr* and *G6pc*) expression. (**C** and **D**) the changes of lipid metabolism genes (*Fans* and *Acaca*) expression. (**E** and **F**) the changes of inflammatory markers (*Tnf-α* and *Ccl2*) expression. The white bar represents scrambled shRNA treated group, and the black bar represents *Tlr4* shRNA treated group. Pklr: pyruvate kinase; G6pc: glucose-6-phosphatase; Fasn: fatty acid synthase; Acaca: acetyl CoA carboxylase, NCD: normal chow diet group; HFD: high fat diet group; n = 7–8 per group. The results are shown as the mean ± S.E.M. ^#^P < 0.05 for HFD group compared with NCD group; *P < 0.05 for *Tlr4* shRNA treated group compared with *scrambled* shRNA treated group.

The expression of several glucose and lipid metabolism-related genes, including pyruvate kinase (*Pklr*), glucose-6-phosphatase (*G6pc*), fatty acid synthase(*Fasn*) and acetyl CoA carboxylase (*Acaca*), in the livers from obese rats were significantly increased(P < 0.05, Fig. 4A–D). After down-regulation of *Tlr4* mRNA expression, these metabolism-related genes expressions in the livers from obese rats were significantly restored (P < 0.05, Fig. 4A–D). However, down-regulation of *Tlr4* mRNA expression did not affect the expression of these glucose and lipid metabolism-related genes in the livers from NCD-fed rats (Fig. 4A–D). In addition, the expression of several inflammatory marker genes, including *Tnf* and *Ccl2*, were significantly increased in the livers from obese rats (P < 0.05, Fig. 4E and F). The obesity-related inflammation in the livers from obese rats was ameliorated after down-regulation of *Tlr4* mRNA expression (Fig. 4E and F). The expression of *Tnf-α*and *Ccl2* in the livers from NCD-fed rats were not affected by down-regulation of *Tlr4* mRNA expression (Fig. 4E and F).

Moreover, Histological analysis revealed that the adipocyte from obese rats displayed hypertrophy compared with that from NCD-fed rats (Fig. 3K and I). The visceral adipose tissue from obese rats was more weight than that from NCD-fed rats (P < 0.05, Table 2). After down-regulation of *Tlr4* mRNA expression, the adipocyte hypertrophy in obese rats was partially reversed (Fig. 3L), and the quantity of visceral adipose tissue from obese rats were also significantly deceased (P < 0.05, Table 2).

### Down-regulation of *Tlr4* mRNA reversed HFD-induced microglia proliferation in the hypothalamic ARC, and had no effect on the morphology of the hypothalamic ARC

**Figure 5:**
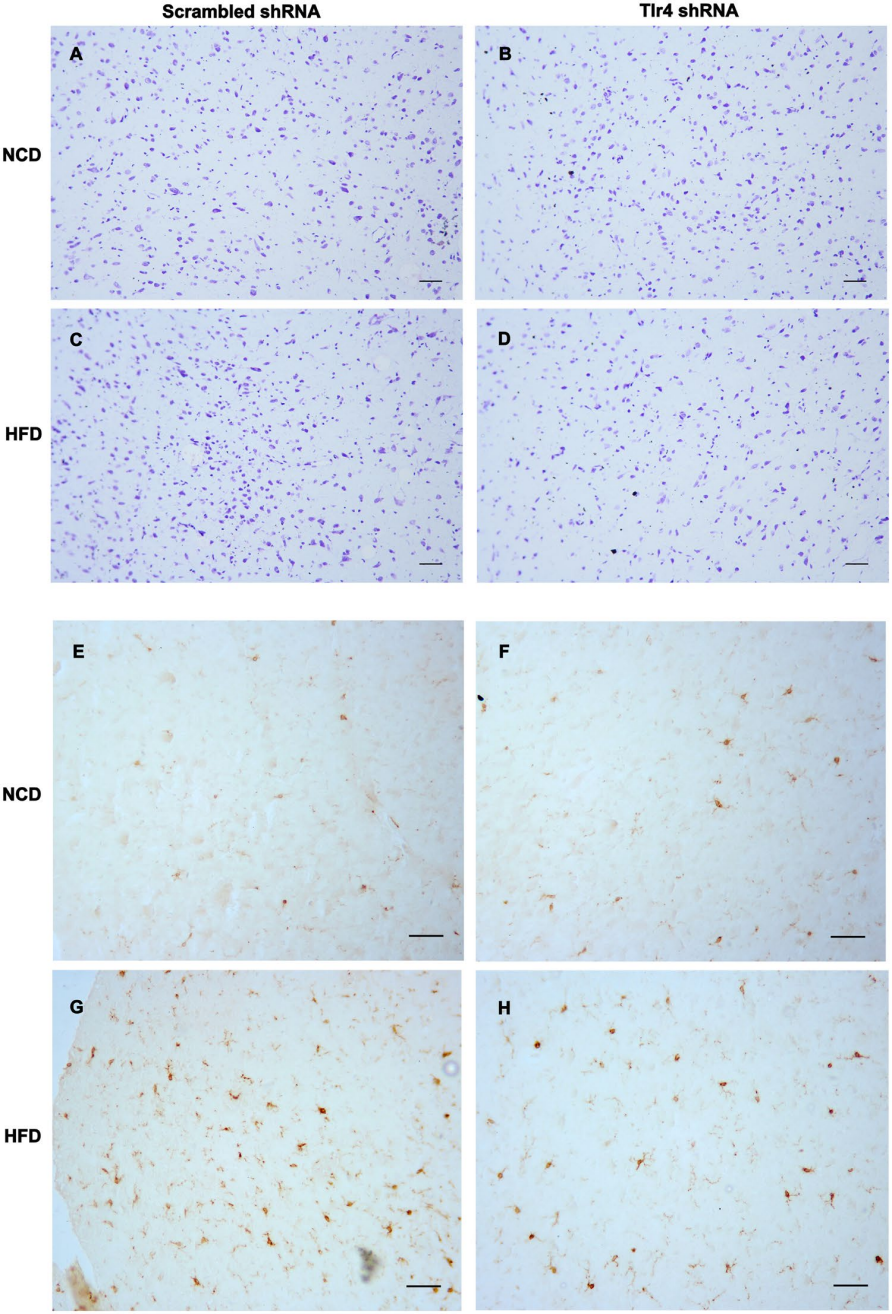
The morphological change of the hypothalamic ARC after down-regulation of *Tlr4* mRNA by lentiviral particles (Nissl’s staining) (**A**–**D**); Down-regulation of Tlr4 mRNA reverses HFD-induced microglia proliferation in the hypothalamic ARC (Microglial cells labeled with Iba1) (**E**–**H**), Scale bars, 100 μm. NCD: normal chow diet group; HFD: high fat diet group.

Immunostaining analysis revealed that *Iba1*-positive cells in the hypothalamic ARC were significantly increased in HFD-fed group than in NCD-fed group (Fig. 5G and E). After down-regulation of Tlr4 mRNA using TLR4 shRNA, the *Iba1*-positive cells were significantly decreased compared with the group using scrambled shRNA (Fig. 5H and G). The TLR4 shRNA treatment had no obvious effect on microglia cells in the hypothalamic ARC (Fig. 5F). Nissl’s staining analysis revealed that down-regulation of Tlr4 mRNA using TLR4 shRNA lentiviral particles did not provoke obvious morphological changes in the hypothalamic ARC (Fig. 5A–D).

## Discussion

In the present study, we used TLR4 shRNA lentiviral particles to suppress the expression of Tlr4 mRNA in the hypothalamic ARC of diet-induced obese rat model by stereotaxic injection. Our results demonstrated that down-regulation of TLR4 expression, which was accompanied by the decreased expression of TNF-α, NPY and increased expression of POMC, in the hypothalamic ARC significantly alleviated diet-induced weight gain and energy intake, and improved peripheral glucose homeostasis, insulin sensitivity and abnormal gluconeogenesis. Moreover, our results found that the high-fat diet-induced hepatic steatosis and adipocyte hypertrophy were also partially reversed after inhibition of TLR4 expression in the hypothalamic ARC, which was accompanied by the expression restoration of several glucose and lipid metabolism-related genes and inflammatory marker genes in the liver. Our data suggest that down-regulation of TLR4 mRNA expression in the hypothalamic ARC is an effective way to improve obesity related metabolic disorders.

In the hypothalamic ARC, TLR4 is mainly expressed on microglia^9, 10, 12^. Activation of TLR4 signaling by dietary saturated fatty acids or LPS can lead to the increased expression of inflammatory cytokines (TNF-α, IL-1 and IL-6) or alteration of activity of NPY/AgRP and POMC neurons^9, 10^. In this study, we found that high-fat diet induced a high level of TLR4 expression in the hypothalamus, which was accompanied by the increased expression of the inflammatory cytokines (TNF-α) and the orexigenic peptide (NPY), and decreased expression of proopiomelanocortin (POMC). Our results are similar with the prior study carried out in the similar obese rat model^13^. These data indicate that not only activation of TLR4 signaling but also up-regulation of TLR4 mRNA expression is involved in the diet-induced hypothalamic metabolic inflammation and dysregulation of arcuate nucleus energy balance circuit. Inhibition of the ligand recognition function of TLR4 by anti-TLR4 antibody or loss-of-function mutation in TLR4 has been proved to reduce hypothalamic inflammation and correct insulin and leptin resistance^10, 11^. Our results further demonstrated that specific down-regulation TLR4 mRNA expression in the hypothalamic ARC also reduced the hypothalamic inflammation (TNF-α). Moreover, it also reduced the expression of NPY and increased the expression of POMC. Taken together, these data indicate that inhibition of TLR4 expression or TLR4 function in the hypothalamic ARC all can reduce diet-induced hypothalamic metabolic inflammation.

Peripheral insulin resistance is contribute to glucose metabolism disorder in obese conditions^14^. High level of fasting glucose, hyperinsulinemia and impairment of glucose and insulin tolerance are the common features of obesity^15, 16^. Our high-fat diet-induced obese rats have the similar features of glucose metabolism disorders. One study demonstrated that inhibition of the function of TLR4 by anti-TLR4 antibody in hypothalamus improved insulin signal transduction in the liver from obese rat and correct abnormal glucose tolerance^11^. Moreover, another study by Kleinridders *et al*. revealed that ablation of the TLR4 adaptor MyD88 in brain ameliorated obesity-related hyperinsulinemia and impairment of glucose and insulin tolerance^17^. After specific down-regulation of TLR4 expression in the hypothalamic ARC by TLR4 shRNA lentiviral particles, similar results were observed in our diet-induced obese rat model. Collectively, these results suggest that TLR4 signaling-mediated hypothalamic inflammation play a crucial role in obesity-related peripheral insulin resistance. Either inhibition of TLR4 expression or blockade of TLR4 signaling pathway in the hypothalamus can improve peripheral insulin resistance, and restore glucose homeostasis during the course of diet-induced obesity.

Hepatic steatosis is the hallmark feature of obesity-related liver diseases, which is usually accompanied with hyperlipidemia, abnormal gluconeogenesis and activities of metabolism-related enzymes^15, 18^. Our findings about the livers in this study are consistent with previous studies. After specific down-regulation of TLR4 expression in the hypothalamic ARC, we found the hepatic steatosis was partially reversed, which along with restoration of the expression of glucose and lipid metabolism-related genes. In addition, the obesity related abnormal gluconeogenesis was also ameliorated. The study by Milanski *et al*. revealed that inhibition of the function of hypothalamic TLR4 by anti-TLR4 antibody reversed obesity-related liver diseases by improved insulin signal transduction in the liver^11^, which is in line with our findings. Furthermore, adipocyte hypertrophy in our obese rats was also partially reversed after inhibition of the hypothalamic TLR4 expression, the similar finding was also observed in the study by Kleinridders *et al*., in which the TLR4 adaptor MyD88 was specifically deleted from brain^17^. Thus, down-regulation of TLR4 expression or blockade of TLR4 signaling pathway in the hypothalamus is sufficient to ameliorate obesity-related liver diseases and adipocyte hypertrophy.

In conclusion, we demonstrate that specific down-regulation of TLR4 expression in the hypothalamic ARC is sufficient to improve glucose and lipid homeostasis during the course of diet-induced obesity by inhibition of hypothalamic inflammation. Our findings confirm the previous opinions that the TLR4 signaling pathway in hypothalamus is an attractive target for treatment of obese conditions, and further suggest that ARC-restricted TLR4 knockdown is a potential strategy to combat metabolic disorders associated with obesity.

## Methods

### Animals

Four-week-old male Sprague-Dawley (SD) rats (100–120 g, n = 40) were purchased from Laboratory Animal Center of Dalian Medical University. Animals were housed doubly with a 12-hour light–dark cycle by artificial light and at a constant room temperature of 20–22 °C. All animals had free access to food and water. All animal procedures and protocols were conducted in accordance with the National Institute of Health Guide for the Care and Use of Laboratory Animals (NIH Publications No. 80-23, Revised 1996) and approved by the Ethical Committee of the Second Hospital of Dalian Medical University.

### Animal Grouping and RNAi protocols

All rats were allowed to acclimate new environment for 1 week, and then randomly divided into two groups: normal chow diet (NCD, 10% Kcal from fat; D12450, Research Diets, Inc. USA) group and high fat diet (HFD, 45% Kcal from fat; D12451, Research Diets, Inc. USA) group (n = 20/group). All rats were fed for 14 weeks. During this time, the body weight was recorded weekly. In the 15th week, the rats in NCD group and HFD group were treated with TLR4 shRNA lentiviral particles or scrambled shRNA lentiviral particles (catalogue number: sc-156001-V and sc-108080, Santa Cruz, USA) by stereotaxic injection as our previously described method^19^. After treatment, the food intake was measured daily and the body weight was recorded weekly for 4 weeks. The copGFP Control Lentiviral Particles (catalogue number sc-108084, Santa Cruz, USA) was used to examine the transduction efficiency in hypothalamic ARC by stereotaxic injection.

### Intraperitoneal glucose tolerance test (ipGTT)

The animals were fasted overnight, and then, glucose was administered intraperitoneally (2 g/kg body weight) between 9 am and 10 am. Blood samples were collected from the cut tip of the tail at 0, 15, 30, 60, 90 and 120 min. The blood glucose concentration was measured using Accu-Chek Active glucometer (Roche Diagnostic, Rotkreuze, Switzerland). The glucose response during the glucose tolerance test was calculated by estimating the total area under the glucose curve.

### Intraperitoneal insulin tolerance test (ipITT)

The animals were fasted for 6 h, and then, Insulin was administered intraperitoneally (0.5 U/kg body weight) between 9 am and 10 am. Blood samples were collected from the cut tip of the tail at 0, 15, 30, 60, 90 and 120 min. The blood glucose concentration was measured using Accu-Chek Active glucometer (Roche Diagnostic, Rotkreuze, Switzerland). The glucose response during the insulin tolerance test was calculated by estimating the total area under the glucose curve.

### Intraperitoneal pyruvate tolerance test (iPTT)

The animals were fasted overnight, and then, pyruvate (Sigma) was administered intraperitoneally (2 g/kg body weight) between 9am and 10am. Blood samples were collected from the cut tip of the tail at 0, 15, 30, 60, 90 and 120 min. The blood glucose concentration was measured using Accu-Chek Active glucometer (Roche Diagnostic, Rotkreuze, Switzerland). The glucose response during the pyruvate tolerance test was calculated by estimating the total area under the glucose curve^11^.

### Serum biochemical and hormonal assays

The blood was taken by heart puncture, and serum was immediately separated through centrifugation (3000 rpm for 10 min) and kept under −80 °C until analysis. The concentrations of Tumor Necrosis Factor α (TNF-α), Interluekin-6 (IL-6), Free fatty acids (FFA), insulin, and leptin in serum were measured using rat ELISA kits (CSB-E11987r, CSB-E04640r, CSB-E08770r, CSB-E05070r and CSB-E07433r, Cusabio Biotech Co., Ltd, China). Glucose, Aspartate Aminotransferase (AST), Alanine aminotransferase (ALT), Triglyceride and Cholesterol were measured using automatic measuring analyzer (Roche Diagnostic).

### Histology and Immunohistochemistry

The liver, visceral adipose tissues (including epididymal adipose tissue) and brain were collected. The liver and adipose tissues were weighed. All tissue blocks were fixed in 4% paraformaldehyde solution further histological analysis. Five-micrometer sections were cut from the paraffin-embedded tissues with microtome. The sections of livers and adipose tissue were stained by regular hematoxylin-eosin methodology for evaluation of liver and adipose histology. The sections of brain were stained by Nissl’s stain method for evaluation of the morphological changes of the hypothalamus. For evaluation of the proliferation of microglia in the ARC, the Iba1 (Marker of microglia cells) (ab178847, Abcam, UK) was stained using established immunohistochemistry staining procedures as described previously^19^.

### Real-time RT-PCR

The hypothalamic tissues were isolated as our previously described method^19^. The mRNA expression of *Tlr4*, *Tnf*, *Npy* and *Pomc* in hypothalamus and the mRNA expression of *Ccl2*, *Tnf* and several metabolism genes(*Pklr*, *G6pc,Fasn* and *Acaca*) in liver were measured by real time PCR on TaKaRa PCR Thermal Cycler Dice Real Time System Lite (Takara Bio, Inc., Japan). Total RNA was extracted using TaKaRa MiniBEST Universal RNA Extraction Kit (Code. 9767, Takara Bio, Inc., Japan). cDNA was synthesized using a primeScript RT reagent Kit with gDNA Eraser kit (TaKaRa Code.RR047) on TaKaRa PCR Thermal Cycler Dice (Takara Bio, Inc., Japan). The PCR reaction mixture in a 25 μl volume contained 12.5 μl 2× SYBR Premix Ex Taq II (Code. RR820, Takara Bio, Inc., Japan), 2 μl RT product, 2 μl Primer F/R (each 10 μM) and 8.5 μl dH_2_O. PCR reaction was run the conditions as follows: 95 °C for 30 sec, then 95 °C for 5 sec and 60 °C for 30 sec, for 40 cycles. The β-actin expression level was quantitated as an internal reference. The sequences of the primer pairs were listed in Table 1.The standard curve was constructed from series dilutions of template cDNA. The relative expression of mRNAs was calculated after normalizing with β-actin.

### Statistical analysis

Statistical analysis was performed using the SPSS Software (version 11; SPSS, Inc., Chicago IL, USA). Two-way, repeated-measure ANOVA followed by two-tailed unpaired student’s T tests were used to analyze energy intake and body weight data after stereotaxical injection of *Tlr4* shRNA lentiviral particles. All of the other data were analyzed by two-way ANOVA followed by two-tailed unpaired student’s t tests. Differences were considered significant if P < 0.05. Data are expressed as the mean ± S.E.M.
